# Transcriptome analysis of rice (*Oryza sativa* L.) shoots responsive to cadmium stress

**DOI:** 10.1038/s41598-019-46684-w

**Published:** 2019-07-15

**Authors:** Lijuan Sun, Jun Wang, Ke Song, Yafei Sun, Qin Qin, Yong Xue

**Affiliations:** 1 Institute of ECO-Environment and Plant Protection, Shanghai Academy of Agricultural Sciences, Shanghai, 201403 China; 2 Shanghai Scientific Observation and Experimental Station for Agricultural Environment and Land Conservation, Shanghai, 201403 China; 3 Shanghai Environmental Protection Monitoring Station of Agriculture, Shanghai, 201403 China; 4 Shanghai Engineering Research Centre of Low-carbon Agriculture (SERLA), Shanghai, 201403 China; 5 Shanghai Key Laboratory of Protected Horticultural Technology, Shanghai, 201403 China; 6 College of Resources and Environmental Sciences, Nanjing Agricultural University, No. 1, Weigang, Xuanwu District, Nanjing 210095 China

**Keywords:** Abiotic, Environmental impact

## Abstract

Cadmium (Cd) is highly toxic to living organisms. This study aimed to elucidate the regulation of gene expression in rice shoots under Cd stress. Rice plants were exposed to 0, 50, 75, 100 μmol/L CdCl_2_ in hydroponic culture for 7 d. Transcriptional changes in rice shoots were examined by transcriptome sequencing techniques. A total of 2197 DEGs (987 up-regulated and 1210 down-regulated) were detected in rice shoots under the exposure of 75 μmol/L CdCl_2_. GO and KEGG enrichment analyses showed that genes encoding auxin-responsive protein IAA and peroxidase were up-regulated, while genes encoding proteins involved in signal transduction, including TIFY family, ERF and bZIP were down-regulated. Abundant ROS related terms were also identified and grouped into significantly differentially expressed GO terms, including oxidoreductase activity, catalytic activity, oxidation-reduction process, confirming the enhanced oxidative stress of Cd. Genes encoding photosystem I reaction center subunit and photosynthetic NDH subunit of luminal location were up-regulated in pathway of energy metabolism, suggesting an interference of photosynthesis by Cd stress. Our results improve the understanding of the complex molecular responsive mechanisms of rice shoots under Cd stress.

## Introduction

As a nonessential heavy metal, cadmium (Cd) exerts highly toxic to living organisms. The exposure of Cd inhibits crop growth and thus reduces their yield through cell proliferation and nitrogen metabolism inhibition as well as photosynthesis rate alteration^1–3^. Cadmium ranks as seventh among the top 20 metal toxins as it commonly released into agricultural soil by human industrial and agricultural activities^4^. Cadmium can be easily taken up by crops and subsequently translocated to the edible organs as Cd has high mobility in soil-plant system^5^. Accumulation of Cd in crops is of great concern in agricultural system. Understanding the process of plant Cd accumulation or molecular stress mechanisms is of critical importance for phytoremediation of Cd contaminated soils.

Rice (*Oryza sativa* L.) is the staple food for nearly half of the world’s population, especially for Asian^6,7^. The irrigation of water into paddy soil makes paddy soil more likely than dry cropland to be polluted by contaminant like heavy metals as rice is usually cultivated under flooding conditions before harvest^8^. Recently, cadmium contamination in paddy soil severely threatens the quality of rice and Cd-contaminated rice becomes a big threaten to human being^9^. It has been reported that the soil-to-grain bio-concentration factors of Cd among 20 rice cultivars ranging from 0.30 to 1.112^10^. Investigating on rice Cd uptake processes and translocate pathways will help reducing the environmental risk of Cd and protecting the food safety.

Many genes that encoding specific membrane transport proteins are involved in metal uptake and transport^11^. It has been reported that OsNramp5 is the major transporter for Cd transport to the inner rice root^12^. The restriction of OsNramp5 facilitated the transportation of Cd to rice shoots^13^. OsIRT1 and OsNramp1 are also potentially involved in Cd process^14^, whereas their contributions were much smaller than that of OsNramp5. After uptake by roots, Cd is then quickly translocated to shoots by xylem. The amount of shoots Cd accumulation has been confirmed to largely depends on xylem-mediated Cd long-distance transport between roots and shoots in rice plants^15^ and OsHMA2 and OsHMA3 were well known to play an important role in this process^16^. Exploring and illuminating the genetic and molecular mechanisms to Cd stress will be of great benefit in improving both crop yield and quality.

RNA-Seq is a recently developed method to transcriptome analysis which uses deep-sequencing technologies, making it possible for the researchers to survey the entire transcriptome in a very high-throughput and quantitative manner^17^. Increasing studies using transcriptome analysis based on Illumina RNA-Seq platform have elucidated Cd stress response in rice and understood their related molecular mechanisms. By conducting genome-wide transcriptome anaylsis, Oono *et al*.^18^ reported that Cd stress signaling regulates the gene expression in drought stress signal pathways in rice. Tan *et al*.^19^ identified 1772 differentially expressed genes (DEGs) that involved in hormone signaling and transcriptional regulation in roots of rice seedling after 1 h of high-Cd stress. Both NACs and WRKYs were up-regulated under Cd stress and all of the 6 Cd-up-regulated ABC transporters were pleiotropic drug resistance protein (PDRs), but all the 6 ZRT/IRT-like proteins (ZIPs) were consistently down-regulated by Cd treatment. RNA-Seq analysis found that many genes identified under high Cd stress can also be found to be responsive to low Cd stress and the transporter genes expression tended to associate with the content of Cd stress in rice plants^20^.

Comprehensive understanding the molecular regulatory mechanisms of Cd uptake, accumulation and detoxification is a fundamental step for effective management and genetic manipulation of Cd accumulation in plants^12,13^. With the development of molecular biology, great progress has been made in elucidating the mechanism of metal stress and in identifying many metal stress responsive genes. However, the regulation of gene expression is a complex process, particularly at the transcriptional level. In this study, we applied a comparative RNA-Seq based approach to identify Cd-responsive DEGs in rice shoots after 7 d of 75 μmol/L Cd^2+^ exposure. A total of 2197 DEGs (987 up-regulated and 1210 down-regulated) were detected in rice shoots under Cd stress. Gene ontology (GO) enrichment analysis was carried out to further characterize the main biological functions of these DEGs in rice shoots under Cd. Furthermore, pathway enrichment analysis was performed to estimate the number of DEGs contained at different levels of the KEGG pathway. The results have provided a basis for further understanding of the molecular mechanism associated with Cd stress in the rice seedlings.

## Results

### Plant growth and Cd accumulation

As shown in Table 1, a significant growth inhibition in rice seedlings has been caused by high concentration of Cd. Low concentration of Cd (50 μmol/L) did not significantly affect the height and dry weight of rice seedlings. However, the height of rice seedlings under higher concentration of Cd stress (75, 100 μmol/L) reduced by 14.6–21.9%, while the dry weight of rice seedlings reduced by 10.1–18.4% when compared to the control.

**Table 1:** The height and dry weight of rice seedlings.

Treatment	Height (cm)	Dry weight (g)
CK	21.9 ± 2.5 a	0.2841 ± 0.006 a
50	20.8 ± 1.2 a	0.2848 ± 0.003 a
75	18.7 ± 0.6 b	0.2555 ± 0.001 b
100	17.1 ± 0.7 b	0.2318 ± 0.003 c

**Figure 1:**
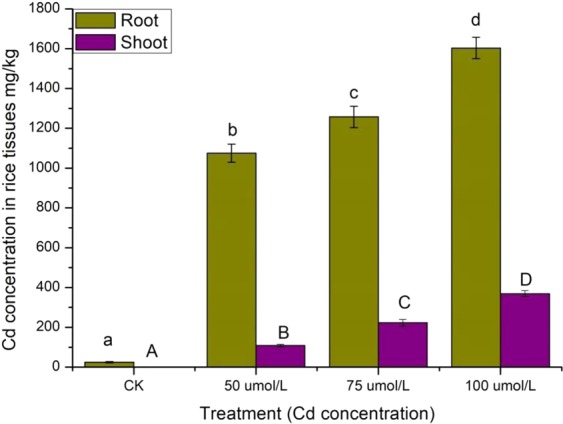
Cd concentration in rice tissues.

The concentration of Cd in different rice roots and shoots was shown in Fig. 1. The concentration of Cd in rice roots was much higher than those of rice shoots, suggesting that Cd accumulated mainly in rice root. The concentration of Cd in both roots and shoots was significantly increased with the concentration of Cd stress.

### Library construction, sequencing and differentially expressed genes (DEGs) identified using RNA-Seq

The rice shoot samples were sampled at the 7 d after treatment with Cd were subjected to total RNA extraction and an RNA-seq analysis. High-throughput sequencing generated 40.92–47.51 million (M) 150 bp paired-end clean reads from the control and Cd stress in rice shoots. After a stringent quality filtering process, 39.73 Gb of clean data (97.76% of the raw data) were obtained, with a Q30 percentage >94%. The counts of clean reads per library ranged from 40.3–46.9 M (Table 2). These findings indicate the good quality of sequencing results. Afterwards, all clean reads were mapped to the rice reference transcriptome. The percentages of mapped reads were similar among the six libraries (91.52–92.09%) (Table 2).

**Table 2:** Summary of RNA-seq data and reads mapping.

Sample	Raw reads	Clean reads Total mapped reads	Q20 (%)	Q30 (%)	GC (%)
CK-1	44764368	44174632 40535581 (91.76%)	97.58	94.02	50.45
CK-2	47293082	46633688 42677098 (91.52%)	97.64	94.12	51.82
CK-3	40924462	40378444 37131465 (91.96%)	97.69	94.23	51.82
Cd-1	45007378	44381150 40782258 (91.89%)	97.72	94.35	51.32
Cd-2	47514004	46934014 43223710 (92.09%)	97.77	94.34	52.37
Cd-3	45455256	44723932 40971004 (91.61%)	97.62	94.19	52.37

**Figure 2:**
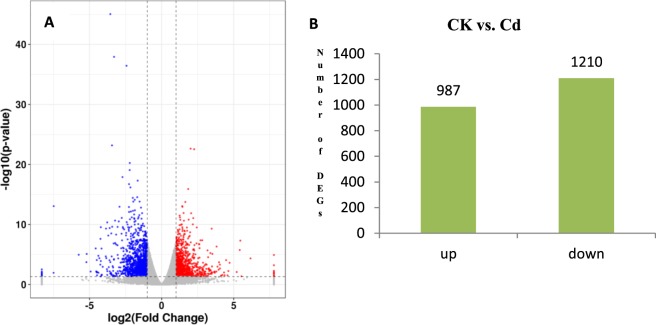
Statistics of differently expressed genes. (**A**) Significantly up‐ or downregulated genes using the threshold of *P* < 0.05 and |log2FoldChange| > 1 in CK vs. Cd. (**B**) Number of upregulated and downregulated transcripts in the treatment of Cd.

The genes with reads per kilobase of transcript per million mapped reads (RPKM) value of six samples were calculated. Differences in gene expression in six samples were examined using the threshold of *P* < 0.05 and |log2FoldChange| > 1. The R package DESeq^21^ was performed to identify the differentially expressed genes. The DEGs were identified by pairwise comparisons of libraries from Cd stress and the control; that is CK vs. Cd. A total of 2197 DEGs were detected in rice shoot under Cd stress, of which 987 DEGs were up-regulated and 1210 DEGs were down-regulated (Fig. 2).

### Validation of RNA sequencing data by quantitative real-time-PCR

To confirm the reliability of sequencing data, a total of 7 differentially expressed genes were selected for quantitative real-time PCR, including heavy metal transport/detoxification protein (*HMTP*), similar to PDR-like ABC transporter (*PDR_ABC*), ATP binding domain containing protein (*ATPB*), ABC transporter G family member 35 (*ABC_TGFM35*), metallothionein-like protein 2B (*MPL2B*), similar to Cadmium tolerant 1 (*CT1*), glycosyl transferase, family 8 protein (*GTF8P*). The results from the qRT-PCR validation for the expression of 7 DEGs showed that the average gene expression levels of *HMTP*, *PDR-ABC*, *ATPB* and *ABC-TGFM 35* were significantly down-regulated in Cd stress as compared to control group, whereas the average gene expression levels of *MPL2B*, *CT1* and *GTF8P* were found significantly up regulated in Cd stress group. As anticipated, the qRT-PCR results were basically consistent with those from RNAseq, suggesting that DEGs resulted from RNAseq was credible for further analysis.

### Functional annotation of DEGs

**Figure 3:**
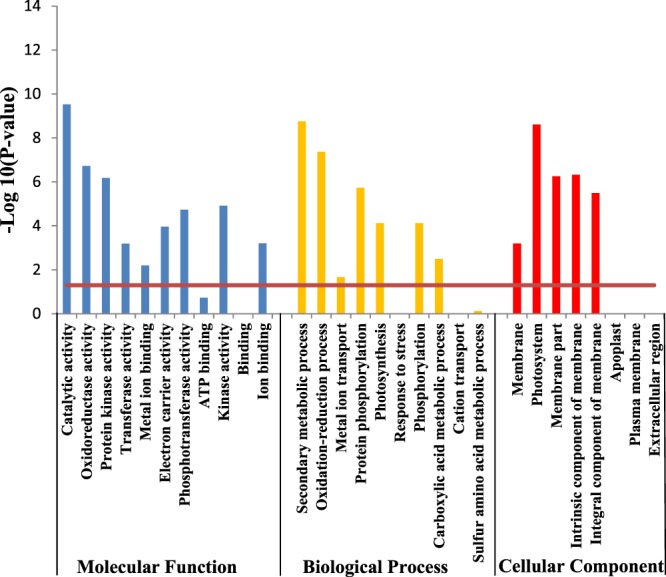
Gene Ontology (GO) enrichment analysis of differentially expressed genes (DEGs) in shoot of rice seedlings. The genes were summarized in three main GO categories (biological process, molecular function and cellular component). *P* value to decide the enrichment significance was calculated through hypergeometric distribution and the *red line* represents the *P* value of 0.05.

Gene ontology (GO) enrichment analysis was carried out to further characterize the main biological functions of DEGs in rice shoots under Cd stress. All the DEGs can be divided into three categories, including molecular function, biological process and cellular component. All the three categories could further be divided into 29 subcategories in shoots, among which 21 subcategories were significantly (*P* < 0.05) enriched under Cd stress (Fig. 3).

In shoots under Cd stress, there were 9, 7 and 5 significantly enriched subcategories belong to molecular function, biological process and cellular component, respectively. Among all the molecular function, the three most significantly enriched subcategories were ‘catalytic activity’, ‘oxidoreductase activity’, ‘protein kinase activity’. Among all the biological process, the three most significantly enriched subcategories were ‘secondary metabolic process’, ‘oxidation-reduction process’, ‘protein phosphorylation’. Among all the cellular component, the three most significantly enriched subcategories were ‘photosystem’, ‘membrane part’ and ‘intrinsic component of membrane’.

### KEGG enrichment analysis of DEGs

**Figure 4:**
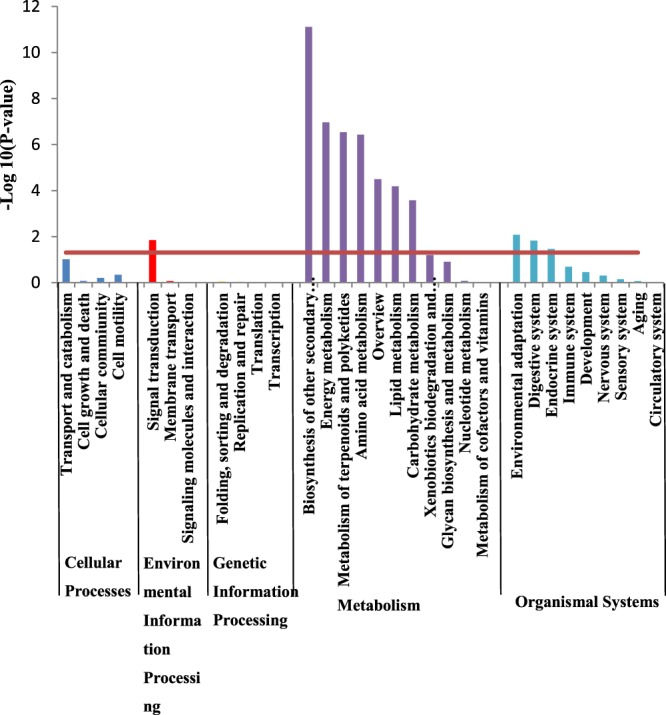
KEGG pathway enrichment analysis of DEGs in shoot of rice seedling between Cd and CK treatment. P value to decide the enrichment significance was calculated through hypergeometric distribution and the red line represents the P value of 0.05.

In order to estimate the number of DEGs contained at different levels of the KEGG pathway, pathway enrichment analysis was performed. Based on the whole genome, the hypergeometric distribution was used to calculate which pathway was significantly enriched of DEGs. There were five KEGG pathway categories, including cellular processes, environmental information processing, genetic information processing, metabolism and organismal systems (Fig. 4).

In shoots under Cd stress, to further identify the possible functional pathway, 776 DEGs were found in 193 KEGG functional pathways by KEGG pathway analysis, which means that some of those DEGs are involved in more than one pathway. Among these pathways, the category of ‘signal transduction’ (20, 10.36%) represented the largest group, followed by ‘carbohydrate metabolism’ (15, 7.77%), ‘lipid metabolism’ (14, 7.25%), ‘amino acid metabolism’ (13, 6.74%) and ‘endocrine system’ (13, 6.77%). The KEGG enrichment analysis showed that only the pathway of ‘signal transduction’ was significantly enriched (*P* < 0.05) in the category of environmental information processing. In category of metabolism, seven pathways including ‘biosynthesis of other secondary metabolites’, ‘energy metabolism’, ‘metabolism of terpenoids and polyketides’, ‘amino acid metabolism’, ‘overview’, ‘lipid metabolism’ and ‘carbohydrate metabolism’ were significantly enriched (*P* < 0.05). In category of organismal systems, three pathways including ‘environmental adaptation’, ‘digestive system’ and ‘endocrine system’ were significantly enriched (*P* < 0.05). However, no enrichment in the category of cellular processes and genetic information processing was found.

There were 189 putative Cd-stress related genes identified in the 11 major significantly enriched pathways, in which 55 genes were up regulated and 134 genes were down regulated (Table S3). In the pathway of signal transduction, four of the up-regulated genes (8) encoding auxin-responsive protein IAA (9, 25, 17 and 24) were found. Five of the down-regulated genes (14) encoding protein TIFY (11d, 11e, 11b, 11c, 9) and two of the down-regulated genes (14) encoding bZIP transcription factor were found. In the pathway of biosynthesis of other secondary metabolites, four of the up-regulated genes (8) encoding Beta-glucosidase (1, 5, 19) and three genes encoding peroxidase were found. Among the 34 down-regulated genes, six genes encoding peroxidase and four genes encoding cinnamyl alcohol dehydrogenase were found. In the pathway of energy metabolism, six genes of the up-regulated genes (13) encoding photosystem I reaction center subunit (VI, psaK, V, III, XI, chloroplastic, O) and two genes encoding photosynthetic NDH subunit of luminal location (3, 2 chloroplastic) were found. In the pathway of metabolism of terpenoids and polyketides, one gene encoding cytochrome P450 family protein was found to be down-regulated. In the pathway of amino acid metabolism, genes related to chloroplastic like chorismate synthase, anthranilate synthase beta subunit 2 and anthranilate phosphoribosyl transferase were found to be down-regulated. In the pathway of overview, up-regulated genes encode chloroplast precursor (cysteine synthase) and fructose-bisphosphate aldolase were found. Down-regulated genes encoding S-adenosylmethionine synthase (1, 2) were found. In the pathway of lipid metabolism, gene encoding acetyl-CoA C-acyltransferase, acyl-coenzyme a oxidase 3, alcohol dehydrogenase 2 and allene oxide synthase were found to be down-regulated. In the pathway of carbohydrate metabolism, genes encoding chloroplastic process like phosphoglucomutase and ribose-5-phosphate isomerase were found to be up-regulated. In the pathway of environmental adaptation, four genes encoding calcium-binding protein were found among the 15 down-regulated genes. In the pathway of endocrine system, one gene encoding acyl-coenzyme a oxidase 4 was found to be up-regulated, while three genes encoding acyl-coenzyme a oxidase 3 and acetyl-coA c-acyltransferase were found to be down-regulated.

## Discussion

It has been reported that most plants exhibit Cd toxicity when the leaves accumulate more than 5–10 mg/kg Cd^22^. In the present study, rice seedlings accumulated 108.24–368.96 mg/kg Cd in the shoot, 24.40–1603.58 mg/kg in the root. A significant inhibition of rice growth has been observed in treatment of 75 and 100 μmol/L Cd^2+^. High Cd concentration exposure has been previously shown to elicit robust physiological responses and gene expression as acute toxic responses in rice seedlings^23,24^. No obvious toxic symptoms can be found in treatment of 50 μmol/L Cd^2+^ in the present rice varieties (Table 1). This result differed from what has been found by Oono *et al*.^20^, which may due to the different treating and exposure time of rice seedlings with Cd stress. The results suggested that high Cd concentration exposure causes fatal damage to rice seedlings.

**Figure 5:**
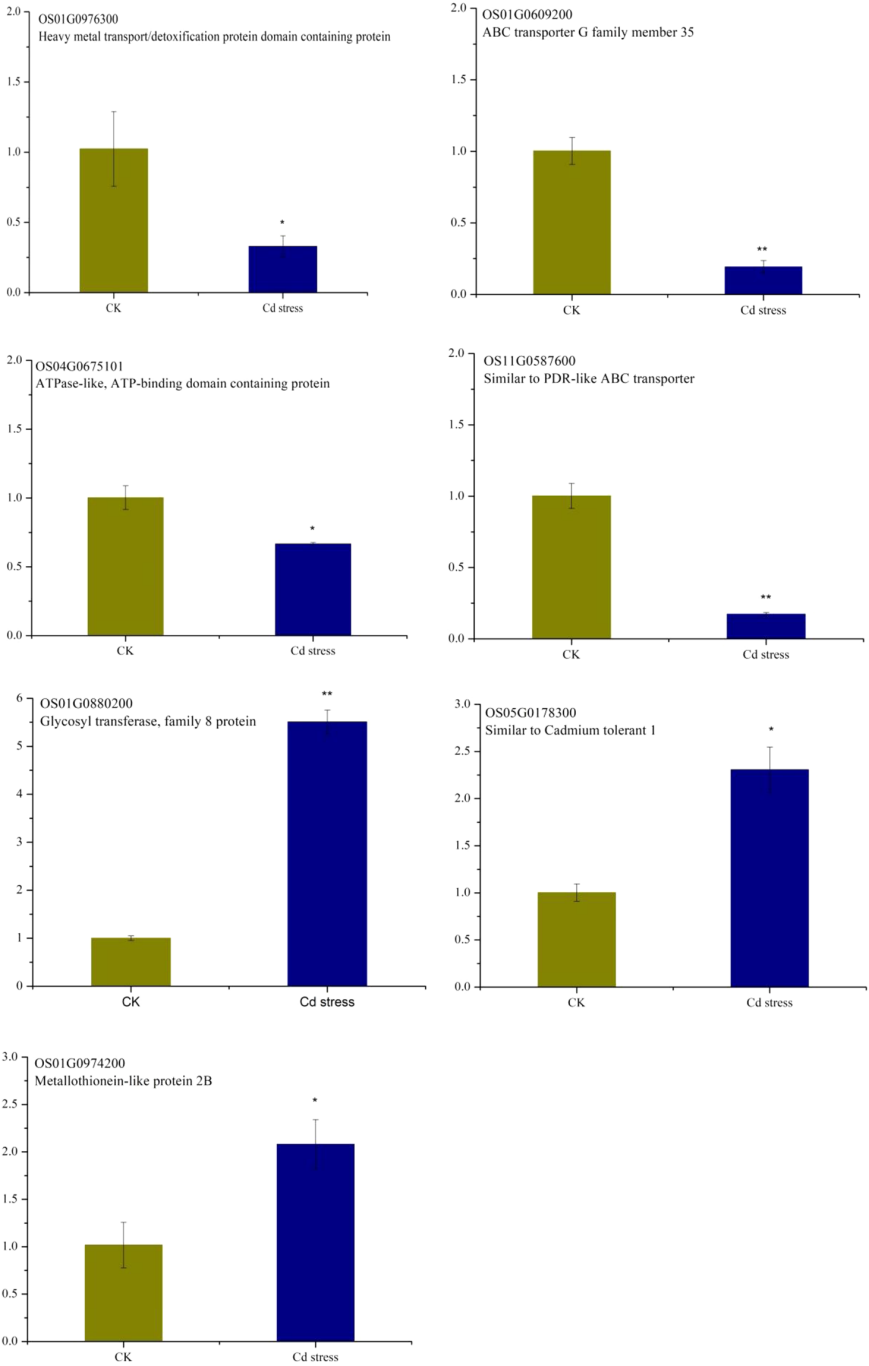
The relative gene expression of 7 randomly selected genes examined by quantitative real-time PCR.

Determining the molecular mechanisms involved in the responses to heavy metal stress would enable researchers to explore the potential heavy metal defensive strategies that may occur in rice plants. Cadmium is a non-essential element, it can actively enter into plant cells by uptake mechanisms for essential elements, such as Zn, Ca and Fe^25^. It is believed that Cd share an entry route with Fe and Mn^12,13^. The high-throughput RNA-Seq analysis allowed us to obtain an overall survey of genes and/or processes potentially related to the early responses in plants to stimuli imposed by changes under abiotic stress. In this study, a total of 2197 DEGs were detected in rice shoot under Cd stress, of which 987 DEGs were up-regulated and 1210 DEGs were down-regulated. The number of up-regulated transcripts ranged from 4529 to 6515, whereas the number of down-regulated transcripts ranged from 2359 to 8734 under 0.2 μM Cd. The number of up-regulated transcripts ranged from 5830 to 7271, whereas the number of down-regulated transcripts ranged from 2965 to 10020 under 1 μM Cd^20^. The transcripts of 1172 genes were regulated after Cd treatments in rice roots, of which Cd-regulated genes related to unfolded protein binding and sulfate assimilation^26^. In our present study, the gene OS01G0974200 that encoding metallothionein-like protein 2B was up-regulated by Cd stress (Fig. 5), which suggested that Cd-regulated genes in rice shoot also related to sulfate assimilation. This phenomenon maybe an implication that rice inherited complex adaptation network in dealing with Cd stress conditions and provides also more candidate genes for further research on rice.

GO enrichment analysis helped showing differentially genes function of the biological process, molecular function, and cellular component involved in the response to various stress condition^27^. Based on GO enrichment analysis, all of these DEGs were mainly distributed in ‘molecular function’, ‘biological process’ and ‘cellular component’ under Cd stress. This indicated that rice initiated broad and complex responsive approaches to adapt to challenges imposed by external Cd stress. Genes related with ‘catalytic activity’ and ‘oxidoreductase activity’ were greatest enriched in the molecular function.

Auxin and its homeostasis play key roles in many aspects of plant growth and development. Previous research has shown that auxin and its transport are involved in plant responses to abiotic stress^28^. In our study, four genes encode auxin-responsive protein IAA (9, 25, 17 and 24) were found to be up-regulated in the pathway of signal transduction in rice shoots under Cd stress (Table 2), which has been confirmed by quantitative real-time PCR (Fig. S1), suggesting that auxin-responsive protein gene was involved in rice response to Cd stress. Yu *et al*.^29^ found that the exogenous exposure of 1-naphthaleneacetic acid improved Cd tolerance in *osaux* 1 mutant and the local auxin gradients provided by auxin transporter are essential for rice Cd tolerance. The auxin physiological response to Cd stress has been partially revealed in *Arabidopsis*^30^. Indole-3-acetic acid (IAA) oxidase activity was also found to be up-regulated after Cd treatment in maize roots and various auxin signaling pathway-related GO terms were significantly enriched in DEGs, which confirmed that auxin affected Cd accumulation in maize seedlings^31^.

The TIFY family is a plant-specific gene family. However, the information about the expression and function of this family in rice is very limited. In the present study, five genes (OS10G0392400, OS10G0391400, OS03G0181100, OS03G0180900, OS04G0395800) encoding TIFY (11 d, 11e, 11b, 11c, 9) were found to be down-regulated in rice shoot under Cd stress, which accounted for 35.7% among the 14 down-regulated genes in the pathway of signal transduction. It has been reported that TIFY (11d, 11e) and TIFY (11c, 11e, 9) were strongly induced by salt and cold, respectively^32^. OsTIFY genes may be involved in the regulation of some common pathways for drought and salt stress responses. Our study suggested that OsTIFY genes may also be involved in the regulation of Cd stress response. Onno *et al*.^18^ reported that TIFY11 was found to be up-regulated in rice root under Cd exposure for 24 h. The difference results may be explained by the different exposure time and rice tissues of Onno’s study and our present study. This information may provide useful clues for functional characterization of important members of the TIFY family in plant development and Cd stress tolerance in rice.

Transcription factors (TFs), such as WRKY, basic leucine zipper (bZIP), ethylene-responsive factor (ERP) and myeloblastosis (MYB) proteins plays an important role in controlling the expression of their specific related genes in response to Cd stress^33^. In our study, one gene encoding ERF (OS03G0860100) and two genes (OS06G0614100, OS02G0194900) encoding bZIP in the pathway of signal transduction were found to be down-regulated under Cd stress (Table S1), which indicate that TFs may be directly involved in Cd response in rice plants.

As a non-redox metal, Cd can indirectly enhance oxidative stress by depleting free radical scavengers, or via the activation of ROS-producing enzymes like NADPH oxidase. In the present study, three genes (OS07G0677400, OS09G0471100, OS07G0676900) encoding peroxidase were found to be up-regulated and six genes (OS01G0963000, OS03G0235000, OS04G0688200, OS05G0162000, OS11G0210100, OS11G0112200) encoding peroxidase were found to be down-regulated in the pathway of biosynthesis of other secondary metabolites, which suggested a significant role for peroxidase in the Cd stress responses. Besides, a large number of ROS related terms were also identified and grouped into significantly differentially expressed GO terms, including oxidoreductase activity (GO:0016491), catalytic activity (GO:0003824), oxidation-reduction process (GO:0055114), confirming the enhanced oxidative stress of Cd. In other species like moso bamboo, the stress of Cd also enhanced the activities of superoxide dismutase and peroxidasen^34^.

Cadmium can interfere with plant photosynthesis by damaging the photosynthetic apparatus, especially by competitively binding to essential calcium site of photosystem 2^35^ and by altering the activities of photosystem 1 and 2^36^. In the present study, six genes (OS05G0560000, OS07G0148900, OS09G0481200, OS03G0778100, OS12G0420400, OS04G0414700) encoding photosystem I reaction center subunit (VI, psaK, V, III, XI, chloroplastic, O) and two genes (OS07G0105600, OS02G0578400) encoding photosynthetic NDH subunit of luminal location (3, 2 chloroplastic) were found to be up-regulated in the pathway of energy metabolism, suggesting an interference of photosynthesis by Cd stress in rice. Cd is shown to damage both of the photosystem 1 and 2 in plants^36^. Ogawa *et al*.^37^ reported that the photosynthetic pathways are immediately inactivated after Cd stress in rice shoots. Plants induce the expression of PsaC gene which encodes for a subunit of photosystem 1 to compensate the decrease in the rate of photosynthesis, thus alleviating the adverse impact of Cd stress on photosynthetic apparatus^38^.

## Conclusion

A comparative RNA-seq based approach was applied to identify Cd-responsive DEGs in rice seedling shoots after 7 d of 75 μmol/L Cd^2+^ stress. A total of 2197 DEGs were identified in rice shoots under Cd stress. Genes encoding auxin-responsive protein IAA were up-regulated, suggesting that auxin was involved in rice shoots response to Cd stress. Genes encoding proteins involved in signal transduction, including TIFY family, transcription factors like ERF and bZIP were found to be down-regulated by Cd stress. Some genes encoding peroxidase were found to be up-regulated while some were also found to be down-regulated in the pathway of biosynthesis of other secondary metabolites, suggesting that peroxidase plays a critical role in rice responsive to Cd stress. Besides, a large number of ROS related terms were also identified and grouped into significantly differentially expressed GO terms, including oxidoreductase activity, catalytic activity, oxidation-reduction process, confirming the enhanced oxidative stress of Cd. Genes encoding photosystem I reaction center subunit and photosynthetic NDH subunit of luminal location were found to be up-regulated in the pathway of energy metabolism, suggesting an interference of photosynthesis by Cd stress in rice. The results have provided a basis for better understanding of the molecular mechanism associated with Cd stress in rice seedlings.

## Methods

### Plant culture and Cd treatment

Cultivar NO. 39 Zhongzao, was chosen as our study material as it was widely grown in east China. The cultivating methods can be referred to our previous study^39^. After growing for thress weeks, rice seedlings were divided into 4 groups, each group was repeated 3 times. The four groups were exposed with 0, 50, 75, 100 μmol/L CdCl_2_, respectively. All plants grew in a controlled growth chamber with a 16 h potoperiod and nutrient solution with or without CdCl_2_ were changed every two days. After treated for 7 days, rice roots and shoots from CK and Cd^2+^ stressed rice plants were sampled and rapidly frozen in liquid nitrogen and then refrigerated at −80 °C for future RNA extraction.

### Measurement of metal concentrations

Dried and homogenized rice tissue powders were digested with concentrated HNO_3_, HF and 30% H_2_O_2_ (5:2:1). All the digests were transferred to a 25 mL volumetric flask and diluted with 1% HCl solution. The concentration of Cd in the digestions was detected by flame atomic absorbance spectrometry (FAAS). Reagent blank as well as a standard reference plant material^39^ were applied to assess the accuracy of analysis procedures for Cd concentrations in rice tissues. The concentration of Cd determined in the reference plant material was in close agreement with the certified value (0.36 ± 0.03 mg/kg) compared with a reference value (0.38 mg/kg).

### Isolation of total RNA, cDNA library construction, and illumina sequencing

Total RNA from shoot samples was isolated separately using the TRIzol Reagent (Invitrogen, Carlsbad, CA, USA) according to the manufacturer’s instructions. The concentration and purity of total RNA in each sample were determined using a NanoDrop spectrophotometer (Thermo Scientific). Three micrograms of RNA were used as input material for the RNA sample preparations. Sequencing libraries were generated using the TruSeq RNA Sample Preparation Kit (Illumina, San Diego, CA, USA). Briefly, mRNA was purified from total RNA using poly-T oligo-attached magnetic beads. Fragmentation was carried out using divalent cations under elevated temperature in an Illumina proprietary fragmentation buffer. First strand cDNA was synthesized using random oligonucleotides and SuperScript II. Second strand cDNA synthesis was subsequently performed using DNA Polymerase I and RNase H. Remaining overhangs were converted into blunt ends via exonuclease/polymerase activities and the enzymes were removed. After adenylation of the 3′ ends of the DNA fragments, Illumina PE adapter oligonucleotides were ligated to prepare for hybridization. To select cDNA fragments of the preferred 200 bp in length, the library fragments were purified using the AMPure XP system (Beckman Coulter,Beverly, CA, USA). DNA fragments with ligated adaptor molecules on both ends were selectively enriched using Illumina PCR Primer Cocktail in a 15 cycle PCR reaction. Products were purified (AMPure XP system) and quantified using the Agilent high sensitivity DNA assay on a Bioanalyzer 2100 system (Agilent). The sequencing library was then sequenced on a Hiseq platform (Illumina) by Shanghai Personal Biotechnology Cp. Ltd^40^. The raw reads were submitted to NCBI Sequence Read Archive (accession number: PRJNA544413)

### Data filtering, mapping of reads and functional annotation

The raw reads were filtered before data analysis to obtain high quality reads for subsequent analysis. The quality of RNA-Seq raw reads were evaluated using FastQC quality control tool (http://www.bioinformatics.babraham.ac.uk/projects/fastqc/), and then were filtered using the Cutadapt software. The processes included removing adapter sequences, trimming the bases with a quality score <Q20 using a 5 bp 3′ to 5′ window, and the reads with a final length less than 50 bp or with unwanted bases were discarded. Afterwards, a reference genome index was established using Bowtie2 software^41^, and the clean reads were mapped to the reference genome (http://www.ensembl.org/index.html) using Tophat2 software^42^.

### Differentially expressed genes (DEGs) enrichment analysis

For analysis of RNA-Seq data, DEGSeq was performed to identify the DEGs from shoot affected by Cd stress compared to control. Before that, the read numbers mapped to each gene were counted using HTSeq, which as the original level of gene expression, and then the levels of gene expression were standardized by Reads Per Kilo bases per Million reads (RPKM). We use the greater than 2-fold change of expression levels and the expression with significant difference (*P* < 0.05) as the condition for screening the significance of DEGs between Cd stress and control samples. Then Gene Ontology (GO) (http://geneontology.org/) and Kyoto Encyclopedia of Genes and Genomes (KEGG) (http://www.genome.jp/kegg) function enrichment analysis were implemented to the DEGs. The term and pathway of differentially genes significant enrichment was calculated using hypergeometric distribution algorithm with wheat Genome Assembly information as the background.

### Validation of gene expression

To validate the RNA-Seq results, seven genes were selected randomly from shoots and then analyzed using quantitative real-time PCR (qRT-PCR). After rice seedlings were treated with or without 75 μM CdCl_2_ solutions of 7 d, the shoots samples were harvested for RNA extraction. Gene-specific primer pairs were designed using Primer 5.0 software (Premier Biosoft International) as shown in Table S1 and plant samples and total RNA isolation were carried out as described above. ACTIN was used as an internal standard^43^. Two micrograms RNA was reverse-transcribed into cDNA using the iScriptTM advanced cDNA Synthesis Kit (Promega, WI, USA) after treated with RNase-free DNase I (Promega, Madison, WI), and the standard curve of each gene was prepared with several dilutions of cDNA. Quantitative real time PCR was performed using a Rotor-Gene 3000 real-time PCR detection system (Qiagen) with SYBR® qPCR Mix (Toyobo, Tokyo, Japan). Quantitative PCR reactions cycling conditions were performed as follows: 95 °C for 2 min, followed by 40 cycles at 95 °C for 15 s, 60 °C for 15 s, and 72 °C for 30 s. The relative expression value of the different genes was calculated using 2^−ΔΔCt^ method^44^. The experiment was performed three biological replicates.

### Statistical analysis

One-way analysis of variance (ANOVA) was performed on the effect of Cd stress on rice height, dry weight, Cd concentration, expression of genes by using Windows-based SPSS 16.0 (SPSS Inc., Chicago, IL, USA). The statistical significance (*P* < 0.05) of differences among values in the treated samples and the control was evaluated by Fisher least significant difference (LSD) test. The data presented are means ± standard errors.

## Supplementary information

Supplementary Information

## References

[1] Huang SQ (2009). Heavy metal-regulated new microRNAs from rice. J Inorg Biochem..

[2] Qiu Z (2016). Characterization of wheat miRNAs and their target genes responsive to cadmium stress. Plant Physiol Bioch..

[3] Zhang F (2002). Response of Antioxidative Enzymes in Cucumber Chloroplasts to Cadmium Toxicity. J Plant Nutr..

[4] Yang XE, Stoffella PJ (2004). Cadmium tolerance and hyperaccumulation in a new Zn-hyperaccumulating plant species (Sedum alfredii Hance). Plant Soil..

[5] Yu H (2006). Cadmium accumulation in different rice cultivars and screening for pollution-safe cultivars of rice. Sci Total Environ..

[6] Du Y (2013). Affects of mining activities on Cd pollution to the paddy soils and rice grain in Hunan province, Central South China. Environ Monit Asseess..

[7] Qian Y (2010). Concentrations of cadmium, lead, mercury and arsenic in Chinese market milled rice and associated population health risk. Food Control..

[8] Huang JH, Hsu SH, Wang SL (2011). Effects of rice straw ash amendment on Cu solubility and distribution in flooded rice paddy soils. J Hazard Mater..

[9] Hu Y (2009). Cadmium toxicity and translocation in rice seedlings are reduced by hydrogen peroxide pretreatment. Plant Growth Regul..

[10] Song WE (2015). Variation of Cd concentration in various rice cultivars and derivation of cadmium toxicity thresholds for paddy soil by species-sensitivity distribution. J of Integr Agr..

[11] Williams LE, Pittman JK, Hall JL (2000). Emerging mechanisms for heavy metal transport in plants. Biochimica Et Biophysica Acta..

[12] Akimasa S (2012). Nramp5 is a major transporter responsible for manganese and cadmium uptake in rice. Plant Cell..

[13] Ishimaru Y (2012). Characterizing the role of rice NRAMP5 in Manganese, Iron and Cadmium Transport. Sci Rep..

[14] Takahashi R (2011). The OsNRAMP1 iron transporter is involved in Cd accumulation in rice. J Exp Bot..

[15] Uraguchi S, Fujiwara T (2012). Cadmium transport and tolerance in rice: perspectives for reducing grain cadmium accumulation. Rice..

[16] Satoh-Nagasawa N (2012). Mutations in rice (Oryza sativa) heavy metal ATPase 2 (OsHMA2) restrict the translocation of zinc and cadmium. Plant Cell Physiol..

[17] Wang Z, Gerstein M, Snyder M (2009). RNA-Seq: a revolutionary tool for transcriptomics. Nat Rev Genet..

[18] Oono Y (2014). Genome-Wide Transcriptome Analysis Reveals that Cadmium Stress Signaling Controls the Expression of Genes in Drought Stress Signal Pathways in Rice. Plos One.

[19] Tan M (2017). Co-expression network analysis of the transcrptomes of rice roots exposed to various cadmium stresses reveals universal cadmium-responsive genes. BMC Plant Biol..

[20] Oono Y (2016). Genome-Wide Transcriptome Analysis of Cadmium Stress in Rice. Biomed Res Int..

[21] Anders S, Huber W (2010). Differential expression analysis for sequence count data. Genome Biol..

[22] White PJ, Brown PH (2011). Plant nutrition for sustainable development and global health. Plant Soil..

[23] Kyunghee L (2010). Comparative proteomic analysis of the short-term responses of rice roots and leaves to cadmium. J Plant Physiol..

[24] Zhang M (2012). Transcriptional profiling in cadmium-treated rice seedling roots using suppressive subtractive hybridization. Plant Physiol Bioch..

[25] Lu LL (2009). Cadmium uptake and xylem loading are active processes in the hyperaccumulator Sedum alfredii. J Plant Physiol..

[26] Lin CY (2013). Comparison of early transcriptome responses to copper and cadmium in rice roots. Plant Mol Biol..

[27] Curci PL (2017). Transcriptomic response of durum wheat to nitrogen starvation. Sci Rep..

[28] Krishnamurthy A, Rathinasabapathi B (2013). Auxin and its transport play a role in plant tolerance to arsenite-induced oxidative stress in Arabidopsis thaliana. Plant Cell & Environ..

[29] Yu CL (2015). The auxin transporter, OsAUX1, is involved in primary root and root hair elongation and in Cd stress responses in rice (Oryza sativa L.). Plant J..

[30] Feng HY (2013). Cadmium interferes with maintenance of auxin homeostasis in Arabidopsis seedlings. J Plant Physiol..

[31] Yue, R. *et al*. Transcriptome Analysis of Cadmium-Treated Roots in Maize (Zea mays L.). *Front Plant Sci*, **7**(1298) (2016). 10.3389/fpls.2016.01298 PMC5006096 27630647

[32] Ye H (2009). Identification and expression profiling analysis of TIFY family genes involved in stress and phytohormone responses in rice. Plant Mol Biol..

[33] Dal Corso G, Farinati S, Furini A (2010). Regulatory networks of cadmium stress in plants. Plant Signal Behav..

[34] Li S (2016). Cadmium-induced oxidative stress, response of antioxidants and detection of intracellular cadmium in organs of moso bamboo (Phyllostachys pubescens) seedlings. Chemosphere..

[35] Hodoshima H (2007). Differential regulation of cadmium-inducible expression of iron-deficiency‐responsive genes in tobacco and barley. Physiol Plantarum..

[36] Siedlecka A, Baszynski T (1993). Inhibition of electron flow around photosystem- I chloroplasts of Cd-treated maize plants is due to Cd-induced iron-deficiency. Physiol Plantarum..

[37] Ogawa I (2009). Time course analysis of gene regulation under cadmium stress in rice. Plant Soil..

[38] Cebeci O (2008). Differential expression of wheat transcriptomes in response to varying cadmium concentrations. Biol Plantarum..

[39] Sun LJ (2017). Mechanism study of sulfur fertilization mediating copper translocation and biotransformation in rice (Oryza sativa L.) plants. Environ Pollut..

[40] Wang J (2019). Morphological and transcriptome analysis of wheat seedlings response to low nitrogen stress. Plants.

[41] Langmead B (2009). Ultrafast and memory-efficient alignment of short DNA sequences to the human genome. Genome Biol.

[42] Trapnell C (2009). Discovering splice junctions with RNA-Seq. Bioinformatics.

[43] Liu Q (2015). Transcriptional and physiological analyses identify a regulatory role for hydrogen peroxide in the lignin biosynthesis of copper-stressed rice roots. Plant Soil..

[44] Kenneth J, Livak TD (2001). Analysis of relative gene expression data using rea l—time quantitative PCR a nd the 2^−Δct^ method. Method..

