# Supplementary material for: Transcriptome analysis of rice (Oryza sativa L.) shoots responsive to cadmium stress
Source: Sci Rep. 2019 Jul 15;9:10177. doi: 10.1038/s41598-019-46684-w (PMC6629703; doi:10.1038/s41598-019-46684-w)
Supplement: Supplementary file 1 — Supplementary Information [file 41598_2019_46684_MOESM1_ESM.pdf]

Transcriptome analysis of rice (*Oryza sativa* L.) shoots responsive to cadmium stress

Lijuan Sun[1, 2, 3, 4, 5], Jun Wang[1, 6], Ke Song[1, 2, 3, 4, 5], Yafei Sun[1, 2, 3, 4, 5], Qin Qin[1, 2, 3, 4, 5*], Yong Xue[1, 2, 3, 4, 5*]

Table S1 Primer information for qRT-PCR

Gene id	Forward (5'-3')	Reverse (5'-3')
actin	GCTTGCATACGTCGCTCTTG	GCTTCCATGCCGATGAGAGA
OS01G0976300	TCCTTGGACATCGACATCGC	CAAGCAGTAAGCAGGGTGGA
OS11G0587600	TGGGCTATCCAGTTGGGAGT	AGGTGAGAGCCCCAAGGAAT
OS04G0675101	ACGAGGAGCCCTGGAGTATT	CCTGGTAGACGAACGACAGG
OS01G0609200	TGCTACTTGCTTTGGCTGGA	CGTGCCGAGAATGAGAGTGT
OS01G0974200	GACCACCTTCCTTGCCGATG	AGCACCAGACCTGCAATCG
OS05G0178300	TAGCCACTGCATATCTTCCAGC	TGTCATCTCCTGGGCACTTG
OS01G0880200	TCTGGGAGAGCATGTTATGGC	GCTACCTTTACAAGCTGTTGCT
OS01G0695800	TAGCGACGAGGGAGCGATAA	TGGGAAGGAATGTGCAAGGT
OS10G0529500	ATGAAGCAGATGTTCGTCGC	AAGCATCCCTTCCCCTTGGA
OS01G0885000	GCATACCTGAAGAACGCCAC	GAGCTGCACATCGTCTCTTC
OS05G0198400	GTCGTGGTGTCGCAGATACT	AGAACTGGTGGAACGAGAGC
OS01G0770700	GGAGGACCGCAAGAACGAC	CATCAAACCAACGTCCACCG
OS01G0612500	TTTGCCCTACTGCACCAACA	TATCATCACGGCCATCCACG
OS01G0842600	GGAGGACCGCAAGAACGAC	CATCAAACCAACGTCCACCG
OS02G0805100	GACCAAACCGGCGAGAAGAA	TGCTCCATCAACTCACGCAT
OS08G0109400	CCACAAAAACCCAACCCACC	TCATCTCGCAGTCCATGTCG
OS05G0230700	GAGCCGCAAGGATGGTGAAT	AACATCGCCAACAAGCATCC
OS07G0182400	TGATGGCTTGTGCTCTTCGT	TCCAGCCTTCGCTCATACAG

Table S2 RNAseq results4 of the 7 selected DEGs

No.	Gene ID	Description	Group	Direction	*P* Value	Fold change
1	OS01G0976300	Heavy metal transport/detoxification protein domain containing protein.	CK vs. Cd	Down	5.14136E-07	0.361742209
2	OS11G0587600	Similar to PDR-like ABC transporter.	CK vs. Cd	Down	4.47683E-08	0.148739417
3	OS04G0675101	ATPase-like, ATP-binding domain containing protein.	CK vs. Cd	Down	0.005668237	0.451707915
4	OS01G0609200	ABC transporter G family member 35	CK vs. Cd	Down	0.000279748	0.26565129
5	OS01G0974200	Metallothionein-like protein 2B.	CK vs. Cd	Up	1.95361E-06	2.119526089
6	OS05G0178300	Similar to Cadmium tolerant 1.	CK vs. Cd	Up	0.000617189	2.22524925
7	OS01G0880200	Glycosyl transferase, family 8 protein.	CK vs. Cd	Up	1.54186E-11	3.669264729
8	OS02G0805100	Auxin-responsive protein IAA9	A vs. B	Up	0.034260966	2.444770548
9	OS08G0109400	Auxin-responsive protein IAA25	A vs. B	Up	8.71948E-14	2.692006093
10	OS05G0230700	Auxin-responsive protein IAA17	A vs. B	Up	7.94477E-10	2.164617854
11	OS07G0182400	Auxin-responsive protein IAA24	A vs. B	Up	0.005182159	2.028183238

Table S3 Eleven major significantly enriched pathways and related genes identified by KEGG enrichment analysis

**Pathway**	**KO**	**Metabolism/gene id**	**Expression under Cd stress**	**Name**	**Annotation**
**Signal transduction**	K14484	OS02G0805100	up	IAA9	Auxin-responsive protein IAA9
	K14509	OS05G0155200	up		Similar to Ethylene receptor.
	K14432	OS01G0813100	up		Similar to FD-like 2 protein (Fragment).
ko04075	K14484	OS08G0109400	up	IAA25	Auxin-responsive protein IAA25
	K14484	OS05G0230700	up	IAA17	Auxin-responsive protein IAA17
	K14431	OS10G0566200	up		Similar to predicted protein.
	K14498	OS04G0432000	up		Serine/threonine-protein kinase SAPK7
	K14484	OS07G0182400	up	IAA24	Auxin-responsive protein IAA24
	K14496	OS05G0473000	Down		Streptomyces cyclase/dehydrase family protein.
	K14488	OS06G0671150	Down		Auxin responsive SAUR protein family protein.
	K13449	OS07G0127500	Down		-
	K13449	OS07G0129300	Down		-
	K14516	OS03G0860100	Down		Similar to Ethylene-responsive transcription factor 2 (Ethylene-responsive element binding factor 2) (EREBP-2) (AtERF2).
	K13464	OS10G0392400	Down		Protein TIFY 11d
	K13464	OS10G0391400	Down		Protein TIFY 11e
	K14431	OS06G0614100	Down		bZIP transcription factor, bZIP-1 domain containing protein.
	K14431	OS02G0194900	Down		Basic-leucine zipper (bZIP) transcription factor domain containing protein.
	K13449	OS07G0127600	Down	S	Allergen V5/Tpx-1 related family protein.
	K13464	OS03G0180800	Down		Similar to ZIM motif family protein.
	K13464	OS03G0181100	Down		Protein TIFY 11b

	K13464	OS03G0180900	Down		Protein TIFY 11c
	K13464	OS04G0395800	Down		Protein TIFY 9
**Biosynthesis of other secondary metabolites** ko00940	K00430	OS07G0677400	Up		Peroxidase.
	K00430	OS07G0676900	Up		Similar to Peroxidase (EC 1.11.1.7).
	K01188	OS05G0365600	Up	G	Beta-glucosidase 19
	K01188	OS01G0930800	Up	G	Beta-glucosidase 5
	K01904	OS10G0578950	Up		Similar to 4-coumarate--CoA ligase-like 2.
	K05350	OS01G0508000	Up	G	Beta-glucosidase 1
	K00430	OS09G0471100	Up		Peroxidase 17
	K01188	OS06G0683300	Up		Similar to Beta-glucosidase.
	K01188	OS04G0474800	Down		-
	K01188	OS04G0474800	Down		Beta-glucosidase 22
	K01188	OS05G0366600	Down		-
	K00083	OS09G0399800	Down	C	Probable cinnamyl alcohol dehydrogenase 8A
	K00083	OS09G0400400	Down	C	Probable cinnamyl alcohol dehydrogenase 8D
	K00083	OS10G0194200	Down	C	Probable cinnamyl alcohol dehydrogenase 1
	K00083	OS02G0187800	Down	C	Cinnamyl alcohol dehydrogenase 2
	K09755	OS10G0512400	Down		
	K01904	OS02G0177600	Down		Similar to 4-coumarate--CoA ligase 1 (EC 6.2.1.12) (4CL 1) (4-coumaroyl-CoA synthase 1) (Clone 4CL14) (Fragment).
	K01904	OS08G0448000	Down		Probable 4-coumarate--CoA ligase 5
	K13064	OS02G0626100	Down		Phenylalanine ammonia-lyase
	K05349	OS03G0749300	Down		-
	K00487	OS05G0320700	Down		Similar to Cytochrome P450.
	K00487	OS02G0467600	Down		Similar to Cinnamate 4-hydroxylase CYP73.
	K13066	OS12G0202700	Down		Similar to N-methyltransferase.

	K09753	OS08G0441500	Down	Similar to Cinnamoyl-CoA reductase.
	K09753	OS09G0127300	Down	NAD(P)-binding domain containing protein.
	K00430	OS01G0963000	Down	Similar to Peroxidase BP 1 precursor.
	K00430	OS01G0327000	Down	-
	K00430	OS03G0235000	Down	Similar to Peroxidase.
	K00430	OS04G0688500	Down	Peroxidase (EC 1.11.1.7).
	K00430	OS04G0688200	Down	Similar to Peroxidase (EC 1.11.1.7).
	K00430	OS04G0688100	Down	-
	K00430	OS05G0162000	Down	Similar to Peroxidase (Fragment).
	K00430	OS08G0113000	Down	-
	K00430	OS10G0109600	Down	-
	K00430	OS11G0210100	Down	Similar to Class III peroxidase 133.
	K00430	OS11G0112200	Down	Similar to Class III peroxidase 136
	K10775	OS02G0627100	Down	Phenylalanine ammonia-lyase
	K10775	OS02G0626400	Down	-
	K10775	OS05G0427400	Down	Similar to Phenylalanine ammonia-lyase.
	K09754	OS05G0494000	Down	-
	K13065	OS04G0500700	Down	Similar to Hydroxyanthranilate hydroxycinnamoyltransferase 3.
**Energy metabolism** ko00195	K02638	OS06G0101600	Up	Plastocyanin, chloroplastic
	K08901	OS07G0544800	Up	Oxygen-evolving enhancer protein 3, chloroplastic
	K08901	OS07G0105600	Up	Photosynthetic NDH subunit of lumenal location 3,chloroplastic
	K08901	OS02G0578400	Up	Photosynthetic NDH subunit of lumenal location 2,chloroplastic
	K02695	OS05G0560000	Up	Photosystem I reaction center subunit VI, chloroplastic
	K02692	OS08G0560900	Up	-
	K02698	OS07G0148900	Up	Photosystem I reaction center subunit psaK, chloroplastic
	K08905	OS09G0481200	Up	Photosystem I reaction center subunit V, chloroplastic

	K02693	OS07G0435300	Up	-
	K02694	OS03G0778100	Up	Photosystem I reaction center subunit III,chloroplastic
	K02699	OS12G0420400	Up	Photosystem I reaction center subunit XI,chloroplastic
	K02701	OS12G0189400	Up	-
	K14332	OS04G0414700	Up	Photosystem I subunit O
	K03541	OS07G0147500	Down	-
	K02641	OS07G0147900	Down	Ferredoxin--NADP reductase, embryo isozyme,chloroplastic
	K02639	OS03G0835900	Down	-
**Metabolism of terpenoids and polyketides** ko00904	K04125	OS05G0560900	Up	Similar to gibberellin 2-beta-dioxygenase.
	K16084	OS02G0569900	Down	Cytochrome P450 family protein.
	K16084	OS06G0599200	Down	Ent-cassadiene C11-alpha-hydroxylase 1
	K16085	OS04G0178400	Down	9-beta-pimara-7,15-diene oxidase
	K14046	OS04G0179700	Down	Syn-pimara-7,15-diene synthase
	K14037	OS12G0491800	Down	Ent-sandaracopimara-8(14),15-diene synthase
	K04120	OS02G0570900	Down	Similar to Ent-copalyl diphosphate synthase 2.
	K04120	OS02G0571100	Down	Terpenoid synthase domain containing protein.
	K04120	OS04G0178300	Down	-
	K14036	OS02G0570400	Down	Ent-cassa-12,15-diene synthase
	K04122	OS06G0569500	Down	Ent-sandaracopimaradiene 3-hydroxylase
	K04122	OS06G0568600	Down	Ent-kaurene oxidase-like 5
	K04121	OS11G0474800	Down	Similar to Isoform 2 of Stemar-13-ene synthase.
**Amino acid metabolism**	K15227	OS06G0708832	Up	-
	K01695	OS07G0182100	Down	-

ko00400	K01609	OS08G0320400	Down	-
	K01609	OS09G0255400	Down	Similar to Indole-3-glycerol phosphate synthase, chloroplast precursor (EC 4.1.1.48) (IGPS).
	K00800	OS06G0133900	Down	-
	K00891	OS06G0225800	Down	-
	K01850	OS01G0764400	Down	Similar to Chorismate mutase, chloroplast precursor (EC 5.4.99.5) (CM-1).
	K15849	OS01G0871300	Down	Bifunctional aspartate aminotransferase andglutamate/aspartate-prephenate aminotransferase
	K01736	OS03G0254800	Down	Chorismate synthase, chloroplastic
	K01658	OS03G0718000	Down	Anthranilate synthase beta subunit 2, chloroplastic
	K00766	OS03G0126000	Down	Anthranilate phosphoribosyltransferase,chloroplastic
	K05359	OS04G0406600	Down	-
	K13832	OS01G0375200	Down	Similar to shikimate biosynthesis protein aroDE.
	K01626	OS07G0622200	Down	Similar to M-160-u1_1 (Fragment).
**Overview** ko01230	K01738	OS01G0814800	Up	Similar to Cysteine synthase, chloroplast precursor (EC 2.5.1.47) (O-acetylserine sulfhydrylase) (O-acetylserine (Thiol)-lyase) (CSase B) (CS-B) (OAS-TL B).
	K01653	OS02G0608550	Up	-
	K01807	OS03G0781400	Up	Probable ribose-5-phosphate isomerase 4,chloroplastic
	K00948	OS03G0100040	Up	-
	K01623	OS06G0608700	Up	Similar to Fructose-bisphosphate aldolase, cytoplasmic isozyme (EC 4.1.2.13).
	K01623	OS12G0169600	Up	Similar to Fructose-bisphosphate aldolase.
	K15227	OS06G0708832	Up	-
	K01609	OS08G0320400	Down	-
	K01609	OS09G0255400	Down	Similar to Indole-3-glycerol phosphate synthase, chloroplast precursor (EC

				4.1.1.48) (IGPS).
K00058	OS08G0447000	Down		Similar to D-3-phosphoglycerate dehydrogenase.
K00800	OS06G0133900	Down		-
K15849	OS01G0871300	Down		Bifunctional aspartate aminotransferase andglutamate/aspartate-prephenate aminotransferase
K00766	OS03G0126000	Down		Anthranilate phosphoribosyltransferase,chloroplastic
K01658	OS03G0718000	Down	E	Anthranilate synthase beta subunit 2, chloroplastic
K00789	OS01G0323600	Down	H	S-adenosylmethionine synthase 2
K00789	OS05G0135700	Down	H	S-adenosylmethionine synthase 1
K00615	OS04G0266900	Down	G	Transketolase C-terminal-like domain containing protein.
K01647	OS02G0194100	Down		-
K00831	OS03G0157900	Down		-
K01623	OS01G0118000	Down		Similar to Fructose-bisphosphate aldolase (EC 4.1.2.13) (Fragment).
K13832	OS01G0375200	Down		Similar to shikimate biosynthesis protein aroDE.
K00873	OS01G0660300	Down		Similar to Pyruvate kinase.
K01626	OS07G0622200	Down		Similar to M-160-u1_1 (Fragment).
K00549	OS12G0623900	Down	E	5-methyltetrahydropteroyltriglutamate--homocysteinemethyltransferase 1
K01695	OS07G0182100	Down		-
K00826	OS03G0231600	Down		Similar to Branched-chain-amino-acid aminotransferase 3, chloroplast precursor (EC 2.6.1.42) (Atbcat-3).
K00891	OS06G0225800	Down		-
K00930	OS02G0657600	Down	E	N-acetyl glutamate kinase 2.
		Down		
		Down		

			Down	
			Down	
			Down	
**Lipid metabolism**	K00454	OS05G0304600	Up	Similar to Lipoxygenase (Fragment).
ko00592	K00232	OS06G0347100	Up	K00232
	K01047	OS03G0708000	Up	Phospholipase A2 homolog 3
	K07513	OS10G0457600	Down	Similar to Acetyl-CoA C-acyltransferase (3-ketoacyl-coa thiolase b) (EC 2.3.1.16) (Fragment).
	K00232	OS06G0354500	Down	Similar to Acyl-coenzyme A oxidase 3, peroxisomal precursor (EC 1.3.3.6) (AOX 3) (Medium-chain acyl-CoA oxidase) (AtCX3).
	K00454	OS08G0509100	Down	Probable lipoxygenase 8, chloroplastic
	K01723	OS03G0225900	Down	Allene oxide synthase 2
	K18857	OS11G0210500	Down	Alcohol dehydrogenase 2
	K10529	OS12G0448900	Down	Alpha-dioxygenase 1
	K01723	OS02G0218700	Down	Allene oxide synthase 3
	K00454	OS08G0508800	Down	Lipoxygenase 7, chloroplastic
**Carbohydrate metabolism**	K01623	OS06G0608700	Up	Similar to Fructose-bisphosphate aldolase, cytoplasmic isozyme (EC 4.1.2.13).
ko00030	K01807	OS03G0781400	Up	Probable ribose-5-phosphate isomerase 4,chloroplastic
	K01835	OS10G0189100	Up	Phosphoglucomutase, chloroplastic
	K00948	OS03G0100040	Up	-
	K03841	OS01G0866400	Up	-
	K03841	OS06G0664200	Up	-
	K01623	OS12G0169600	Up	Similar to Fructose-bisphosphate aldolase.
	K00036	OS02G0600400	Down	Similar to Glucose-6-phosphate dehydrogenase.
	K00615	OS04G0266900	Down	Transketolase C-terminal-like domain containing protein.

	K00033	OS06G0111500	Down		6-phosphogluconate dehydrogenase, decarboxylating 1
	K01623	OS01G0118000	Down		Similar to Fructose-bisphosphate aldolase (EC 4.1.2.13) (Fragment).
**Environmental**	K18835	OS08G0499300	Up		-
**adaptation**	K13413	OS06G0191300	Up		Similar to Mitogen-activated protein kinase kinase 4 (Fragment).
ko04626	K02183	OS09G0458800	Up		EF hand domain containing protein.
	K13447	OS01G0360200	Up		Respiratory burst oxidase homolog protein B
	K13448	OS05G0147300	Up		Probable calcium-binding protein CML22
	K13448	OS01G0955100	Down		Similar to Calmodulin-like protein (Fragment).
	K13448	OS03G0331700	Down		Probable calcium-binding protein CML27
	K13448	OS05G0312600	Down	T	Probable calcium-binding protein CML21
	K13448	OS08G0558100	Down		Probable calcium-binding protein CML48
	K13448	OS08G0558100	Down		Similar to Polcalcin Phl p 7 (Calcium-binding pollen allergen Phl p 7) (P7).
	K13448	OS11G0141400	Down		-
	K13448	OS12G0603800	Down	DTZ	Calmodulin-like protein 5
	K13448	OS12G0138050	Down		-
	K13449	OS07G0127500	Down		-
	K13449	OS07G0127600	Down	S	Allergen V5/Tpx-1 related family protein.
	K13449	OS07G0129300	Down		-
	K13412	OS03G0788500	Down		-
	K13459	OS01G0956800	Down		Hypothetical conserved gene.
	K13459	OS04G0514600	Down		-
	K13457	OS06G0698812	Down		Disease resistance protein domain containing protein.
**Digestive system**	K14685	OS06G0560000	Up		Solute carrier family 40 member 1
ko04978	K00522	OS11G0106700	Down		-
	K00522	OS12G0106000	Down		-
**Endocrine system**	K00232	OS06G0347100	Up		Similar to Acyl-coenzyme A oxidase 4, peroxisomal (EC 1.3.3.6) (AOX 4)

ko03320					(Short- chain acyl-CoA oxidase) (SAOX) (AtCX4) (G6p) (AtG6).
	K00232	OS06G0354500	Down		Similar to Acyl-coenzyme A oxidase 3, peroxisomal precursor (EC 1.3.3.6) (AOX 3) (Medium-chain acyl-CoA oxidase) (AtCX3).
	K07513	OS10G0457600	Down		Similar to Acetyl-CoA C-acyltransferase (3-ketoacyl-coa thiolase b) (EC 2.3.1.16) (Fragment).
	K01897	OS06G0158000	Down		Similar to Acyl-CoA synthetase (EC 6.2.1.3).
	K01897	OS01G0681200	Down	I	-

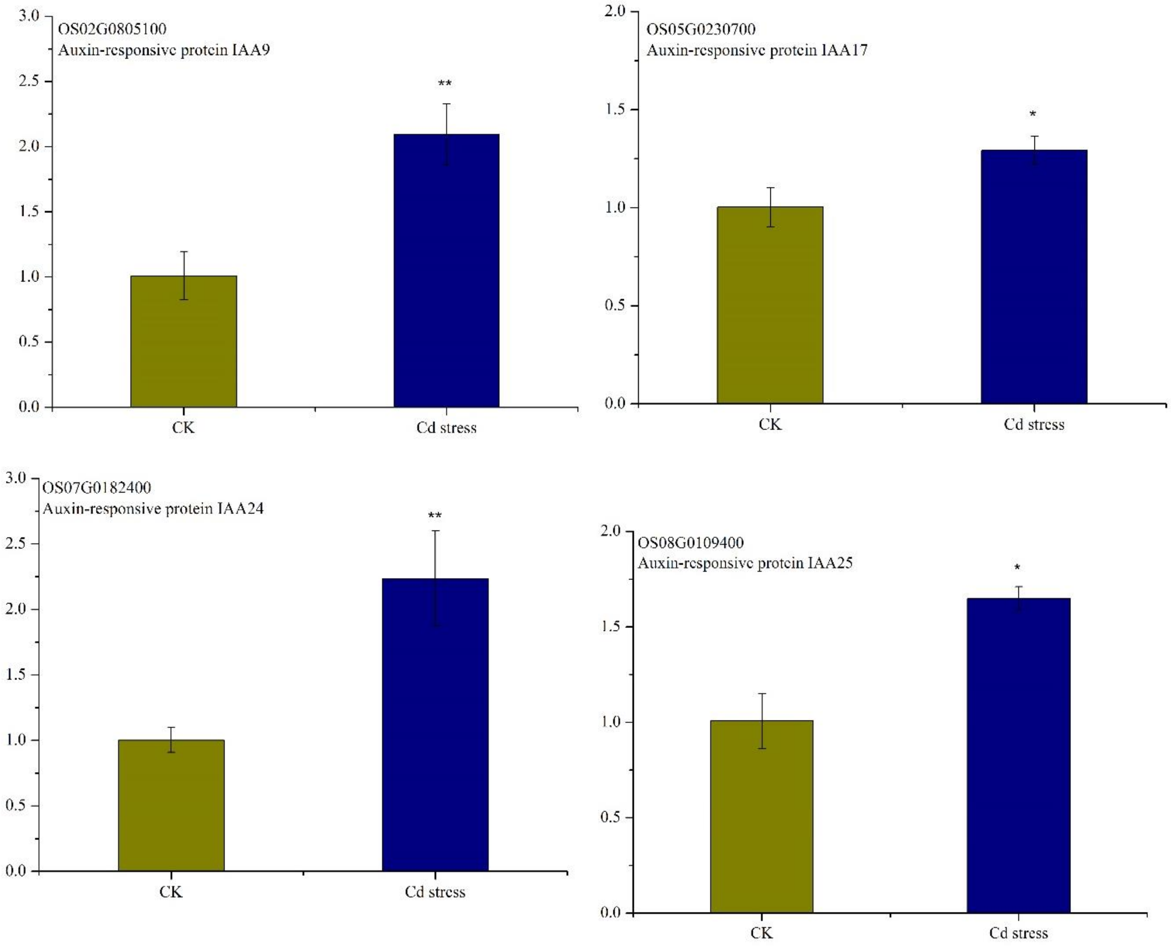

Figure S1 The relative gene expression of 4 auxin-responsive protein genes examined by quantitative real-time PCR
